# Supplementary figures and images for: Causal relationship between several autoimmune diseases and renal malignancies: A two-sample mendelian randomization study
Source: PLoS One. 2024 Feb 29;19(2):e0297861. doi: 10.1371/journal.pone.0297861 (PMC10903885; doi:10.1371/journal.pone.0297861)

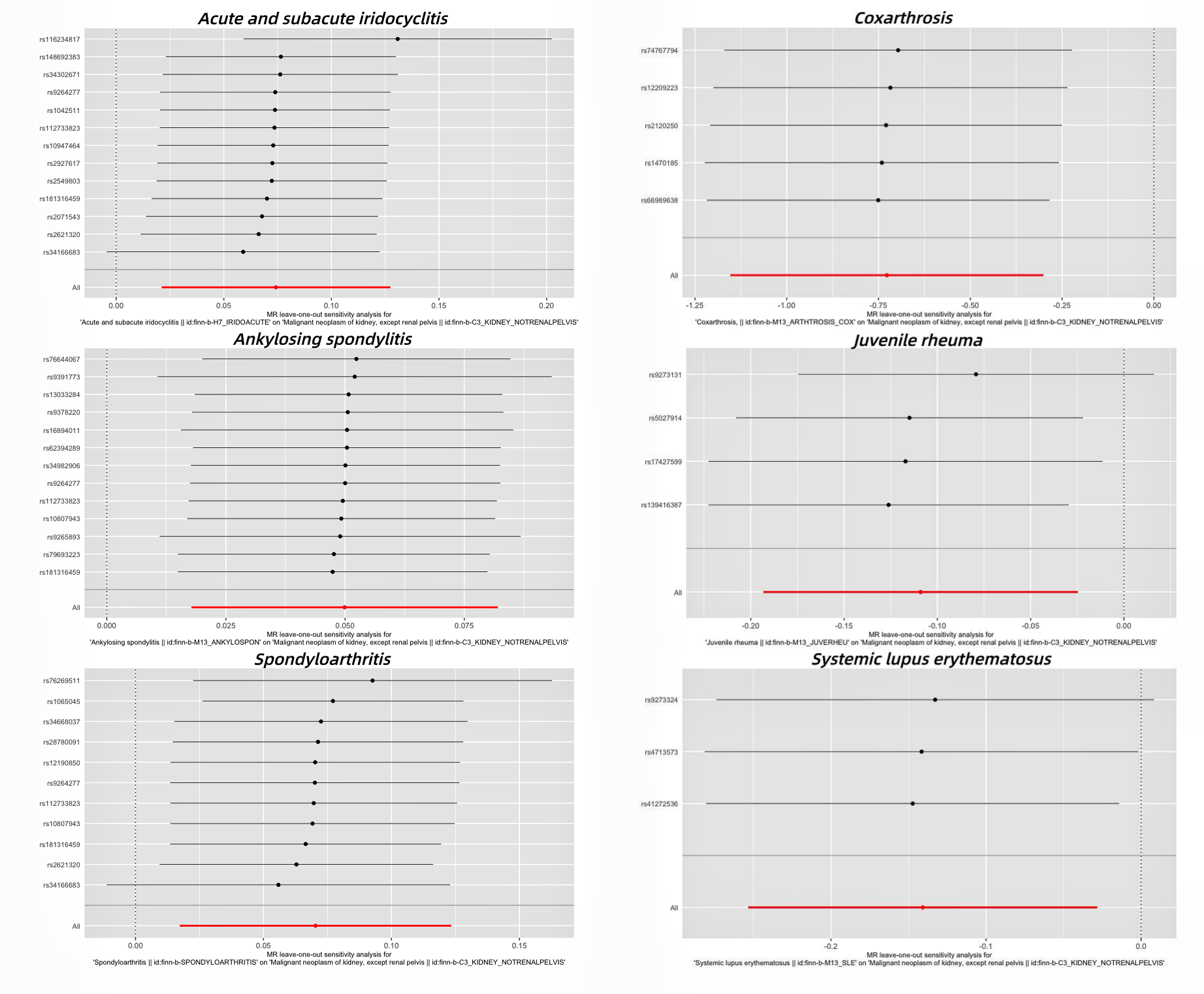

Supplement: S1 Fig — (TIF) [file pone.0297861.s001.tif]

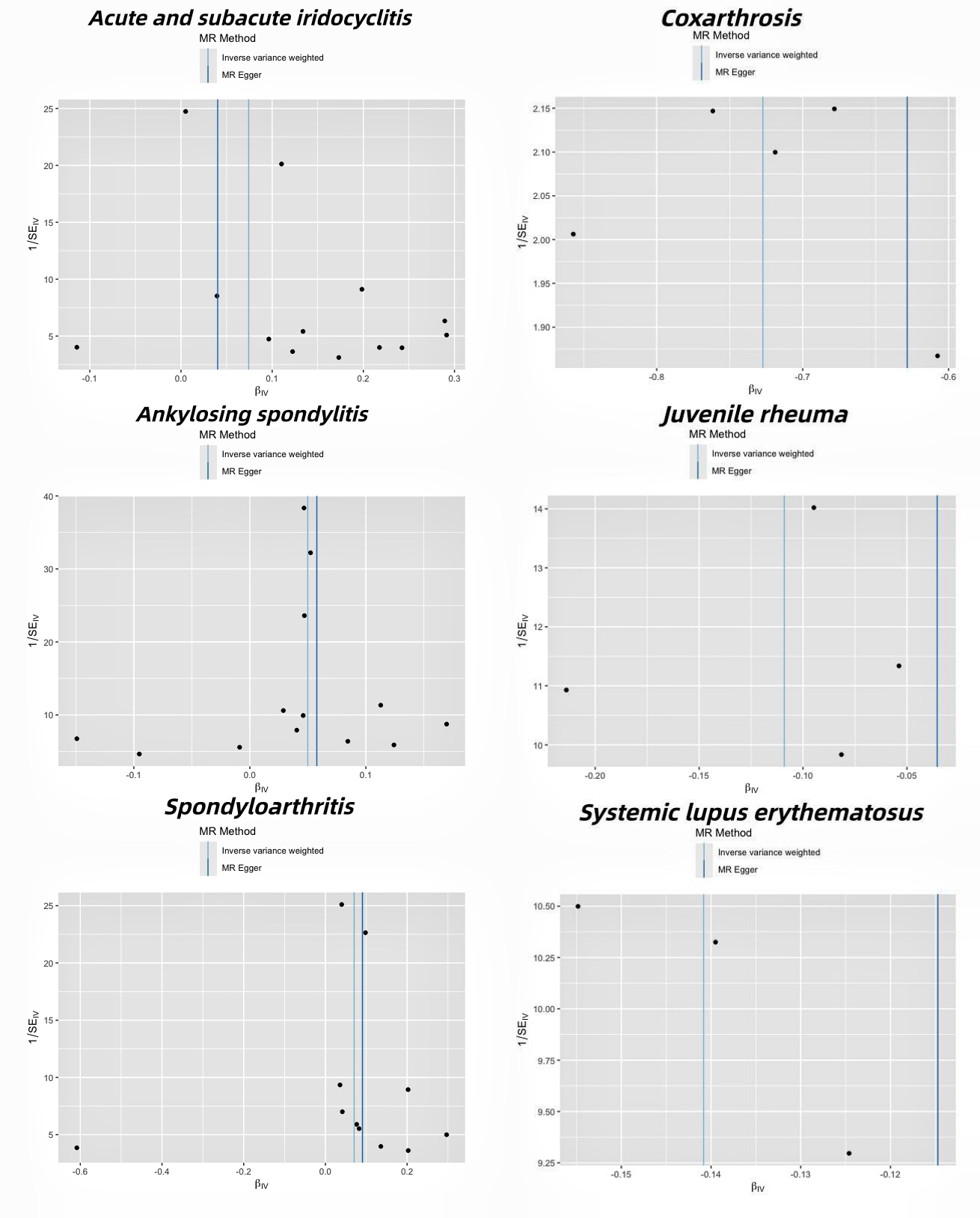

Supplement: S2 Fig — (TIF) [file pone.0297861.s002.tif]

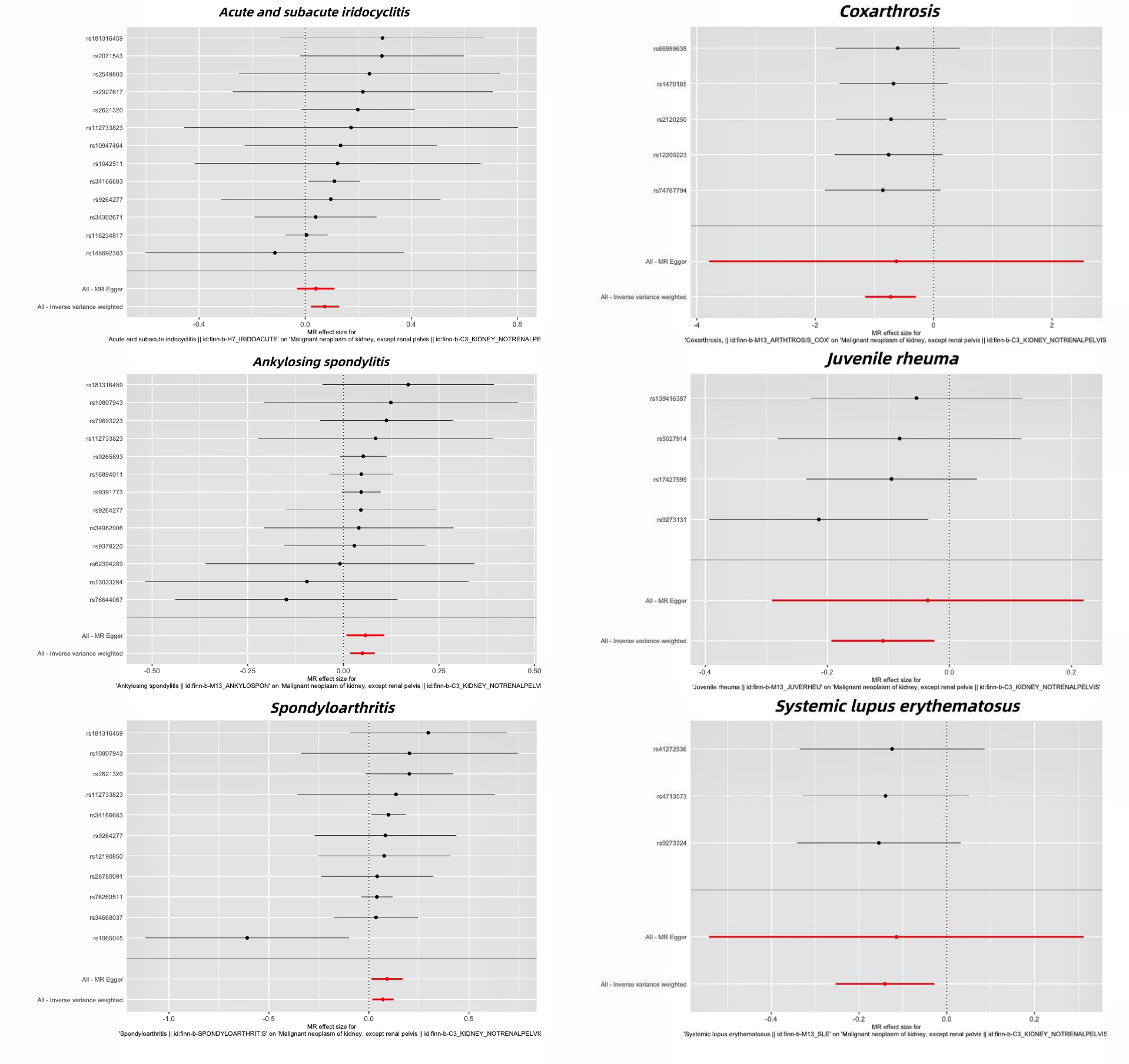

Supplement: S3 Fig — (TIF) [file pone.0297861.s003.tif]

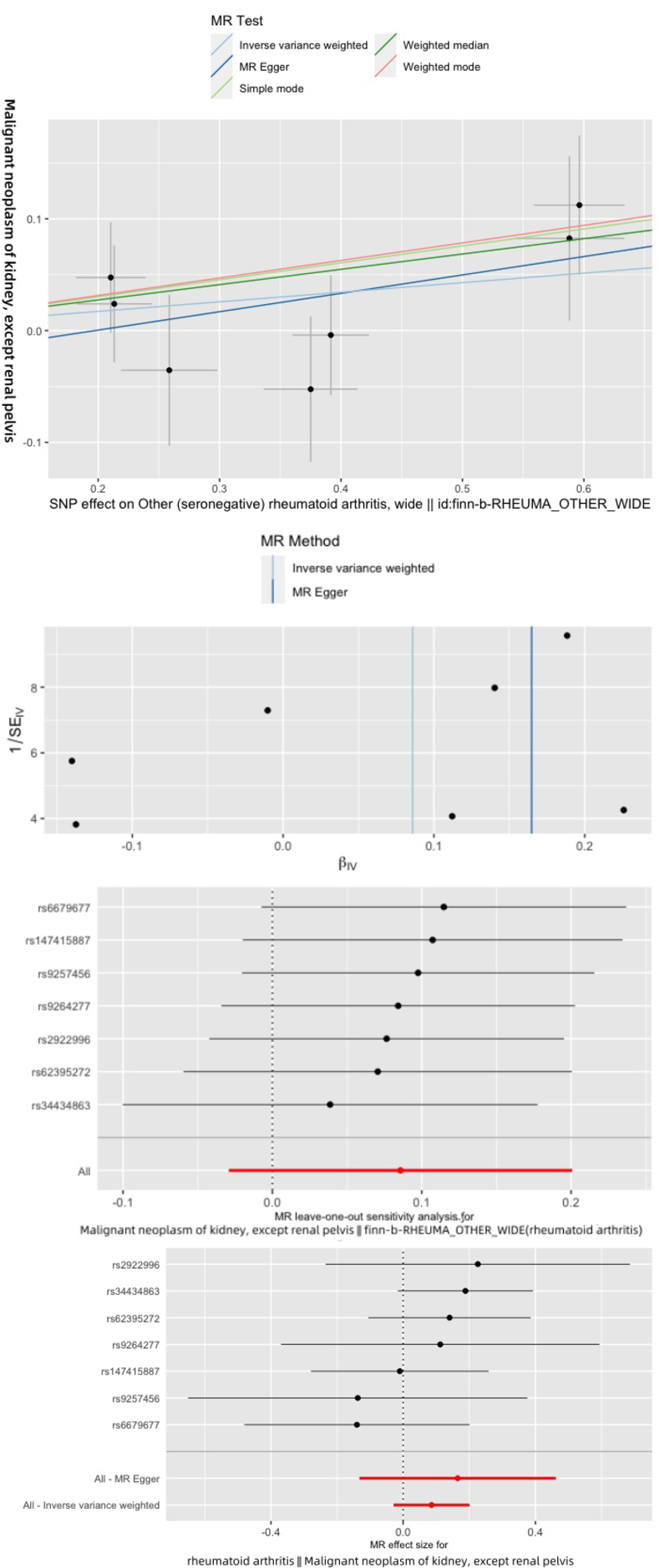

Supplement: S4 Fig — (TIF) [file pone.0297861.s004.tif]
